# Factors affecting the early efficacy of intravenous thrombolysis using alteplase in acute ischemic stroke: A retrospective analysis of 255 cases

**DOI:** 10.1097/MD.0000000000045176

**Published:** 2025-10-17

**Authors:** Jiwei Cheng, Zhen Yuan, Yunqing Zeng, Yangbo Hou, Yunyun Zhang

**Affiliations:** a Department of Neurology, Yueyang Hospital of Integrated Traditional Chinese and Western Medicine, Shanghai University of Traditional Chinese Medicine, Shanghai, China; b Department of Neurology, Putuo Hospital, Shanghai University of Traditional Chinese Medicine, Shanghai, China.

**Keywords:** acute ischemic stroke, alteplase, influencing factors, intravenous thrombolysis, recombinant tissue plasminogen activator

## Abstract

The early efficacy of thrombolysis within 24 hours can accurately reflect its effectiveness, as it is less influenced by other factors. This study aimed to analyze the factors influencing the early efficacy of intravenous thrombolysis using recombinant tissue plasminogen activator (IV-rtPA). The clinical data of 225 acute ischemic stroke patients treated with IV-rtPA from December 2018 to June 2023 were analyzed retrospectively. Univariate analysis and multivariate logistic regression analysis were used to compare data between 2 groups. The effective group included 127 patients (49.8%), while the noneffective group had 128 patients (50.2%). Patients with a baseline National Institute of Health Stroke Scale (NIHSS) score below 10 and those who received IV-rtPA within 3 hours of acute ischemic stroke onset-to-treatment time (OTT) showed greater benefits (NIHSS, χ² = 110.434, *P* < .001; OTT, χ² = 23.196, *P* < .001). Univariate analysis revealed significant differences between the groups in gender (*P* = .030), age (*P* < .001), AF history (*P* = .024), drinking history (*P* = .026), FIB level (*P* = .012), SBP level (*P* = .046), OTT time (*P* < .001), baseline NIHSS (*P* < .001), and rtPA dose (*P* = .012). Multivariate logistic regression analysis identified male gender (OR: 3.986, 95% CI: 1.727–9.201, *P* < .001), drinking history (OR: 0.253, 95% CI: 0.124–0.671, *P* = .004), OTT (OR: 0.958, 95% CI: 0.948–0.969, *P* < .001), baseline NIHSS scores (OR: 0.838, 95% CI: 0.785–0.895, *P* < .001), and rtPA dose (OR: 385.527, 95% CI: 16.725–8886.741, *P* < .001) as independent factors influencing early efficacy. Male gender, drinking history, baseline NIHSS score, OTT, and rtPA dose are independent factors affecting early thrombolysis efficacy. Age, AF history, high FIB level, and high SBP may also be potential risk factors.

## 1. Introduction

As global aging increases, stroke has become one of the leading causes of death and disability worldwide, particularly in China. Studies indicate that the lifetime risk and disease burden of stroke in China reach up to 39.3%, the highest globally.^[1,2]^ Among the different types of strokes, acute ischemic stroke (AIS) is the most prevalent, accounting for 81.9% of all stroke cases. This type of stroke occurs when a blood clot obstructs a blood vessel supplying blood to the brain, leading to significant morbidity and mortality.^[3]^ Despite extensive research, an ideal treatment for AIS remains elusive, and many patients suffer from long-term disabilities. This condition not only drastically impacts their quality of life but also places a heavy burden on their families and society. Effective management of AIS is critical to reduce the incidence of stroke-related disabilities and improve patient outcomes.

One of the most promising treatments for AIS is thrombolysis using intravenous recombinant tissue plasminogen activator (IV-rtPA),^[4]^ for instance, alteplase. Alteplase is a serine protease that aids in the breakdown of blood clots by converting plasminogen to plasmin, the primary enzyme responsible for clot dissolution.^[5,6]^ Approved by the FDA in 1996 for the treatment of AIS, alteplase has become a cornerstone in the management of this condition. Alteplase works by restoring blood flow to the brain, thereby minimizing the extent of brain damage and improving neurological function. Clinical trials and studies have shown that thrombolysis with alteplase can significantly enhance the daily living abilities of patients with AIS.^[7,8]^ However, its efficacy varies widely among patients due to several influencing factors.^[9]^

The time window for administering alteplase is crucial; it is most effective when given within 4.5 hours of stroke onset.^[10]^ Delays in treatment can significantly reduce its benefits and increase the risk of adverse effects, such as intracranial hemorrhage. Additionally, patient-specific factors such as age, baseline stroke severity, and preexisting health conditions can influence the outcomes of alteplase treatment. For example, younger patients and those with less severe strokes tend to respond better to thrombolysis.^[11]^ Despite these challenges, alteplase remains the most effective noninvasive treatment for AIS currently available. It is widely used in clinical practice to improve patient outcomes and reduce the long-term impact of stroke. However, the variability in its efficacy highlights the need for further research to identify additional factors that may influence treatment outcomes.^[12]^ Understanding the factors that influence the efficacy of alteplase is essential for optimizing its use and developing more effective treatment protocols.

In order to further explore the factors that influenced the alteplase effect, the current study retrospectively analyzed the clinical outcomes and its influencing factors of 255 patients who underwent alteplase, and attempted to summarize the potential factors related to the treatment outcomes of the drug.

## 2. Methods

### 2.1. Patients

A total of 255 patients suffering from AIS who underwent IV-rtPA treatment were enrolled in this study at the Department of Neurology, Putuo Hospital, Shanghai University of Traditional Chinese Medicine, from December 2018 to June 2023. The study received approval from the Ethics Committee of Shanghai Putuo District Central Hospital (PTEC-A-2023-11-1). The inclusion criteria for patients were as follows: a clinical diagnosis of AIS confirmed by cranial CT or MRI; receipt of IV-rtPA treatment; complete and accurate clinical data available for analysis; informed consent obtained from the patient or their family members.

Exclusion criteria included: incomplete medical records; multiple complications affecting the analysis; inability to obtain informed consent from the patient or their family members; intracranial hemorrhage or a history of intracranial hemorrhage; significant head trauma or stroke within the past 3 months; presence of an intracranial tumor or large intracranial aneurysm; recent (within 3 months) intracranial or intraspinal surgery or major surgery within the past 2 weeks; gastrointestinal or urinary bleeding within the last 3 weeks; active visceral bleeding or aortic arch dissection; artery puncture at a site difficult to compress within the past week; elevated blood pressure (systolic ≥ 180 mm Hg or diastolic ≥ 100 mm Hg); acute bleeding tendencies, including platelet count < 100 × 10^9^/L; administration of heparin within 24 hours; administration of oral anticoagulants with an INR > 1.7 or PT > 15 seconds; administration of thrombin inhibitors or Xa inhibitors within 48 hours, or abnormal sensitive laboratory indices (e.g., APTT, INR, platelet count, ECT, TT, or factor Xa activity assay); blood glucose levels < 2.8 mmol/L or > 22.2 mmol/L; large infarctions on head CT/MRI scan (area of low-density shadow > 1/3 of the cerebral hemisphere).

### 2.2. Thrombolytic drug and administration method

The thrombolytic drug used in this study was Actilyse (Alteplase for Injection, Boehringer Ingelheim Ltd., Germany, lot number: S20160054). The dose was 0.9 mg/kg with a maximum dose of 90 mg. Of the total amount, 10% was completed in 1 minute by intravenous injection and the rest was administered for 1 hour by intravenous drip.

### 2.3. Grouping

The modified Rankin Scale (mRS) score is typically used to evaluate the effect of thrombolysis 3 months post-treatment. However, the mRS score at 3 months can be influenced by various treatments administered after the initial 24 hours, including medications and rehabilitation, which can vary significantly among patients. In contrast, the effect of thrombolysis within the first 24 hours is less likely to be influenced by other treatments, as minimal additional interventions are administered during this period. Furthermore, studies have shown a positive correlation between the effect at 24 hours and the effect at 3 months.^[13]^

Therefore, we used early significant improvement in neurological function 24 hours after thrombolysis as the criterion for effective thrombolysis, dividing the cases into effective and noneffective groups. Early significant improvement in neurological function was defined, based on previous studies, as a decrease in the National Institute of Health Stroke Scale (NIHSS) score by more than 4 points or the complete resolution of all neurological impairments 24 hours after thrombolysis.^[14]^ Additionally, we assessed the mRS scores of patients in both groups 90 days after thrombolysis. An mRS score of 0 to 1 was classified as an excellent outcome, an mRS score of 0 to 2 as functional independence, and an mRS score of 3 to 6 as a poor prognosis.

### 2.4. Data collection

This study collected the following potential factors influencing the early clinical effect of thrombolysis: gender, age, history of previous diseases (hypertension, diabetes, stroke, coronary heart disease, atrial fibrillation), liver dysfunction, smoking history, drinking history, use of antiplatelet drugs history, use of antihypertensive drugs history, use of hypolipidemic drugs history, use of hypoglycemic drugs history, stroke subtypes, fibrinogen level prior to thrombolysis, blood glucose levels prior to thrombolysis, systolic and diastolic blood pressure prior to thrombolysis, NIHSS score at baseline and 24 hours after thrombolysis, low-density lipoprotein level, homocysteine level, onset-to-treatment time (OTT), and the dose of the thrombolytic drug. The data were collected and organized using an Excel spreadsheet, and compared with some important previous studies as context only, including The National Institute of Neurological Disorders and Stroke part I (NINDS I),^[15]^ The National Institute of Neurological Disorders and Stroke part II (NINDS II),^[16]^ The European Cooperative Acute Stroke Study (ECASSIII),^[17]^ and Safe Implementation of Thrombolysis in Stroke-Monitoring Study (SITS-MOST).^[18]^

### 2.5. Statistical analysis

The classification variables were expressed as frequency (n) and percentage (%). The Chi-square test or Fisher exact test was used for classification variables. Continuous variables were expressed as mean ± standard deviation or median (interquartile range) [M (P25, P75)]. If the data met the normal distribution and homogeneity of variance, an independent samples *t* test was used for comparisons between groups; otherwise, the Mann–Whitney *U* test was performed. Initially, a univariate analysis was conducted, followed by a binary multivariate logistic regression analysis for independent variables with statistically significant differences. Candidate predictors for univariate screening were chosen a priori based on: established clinical relevance in AIS thrombolysis studies^[19,20]^ and availability in our dataset. Each variable was tested in univariate logistic regression, and those with *P* < .10 were entered into the multivariate model to balance inclusion of potentially important predictors with model parsimony. Statistical analysis was performed using the Statistical Package for the Social Sciences (SPSS) Software (Version 20.0, IBM, Chicago). A *P*-value of <.05 was considered statistically significant.

## 3. Results

### 3.1. Baseline information

A total of 255 patients were included in current study. Table 1 shows the baseline characteristics of patients enrolled in the current study and some other important studies (NINDS I, NINDS II, ECASSIII, SITS-MOST). There were 127 (49.8%) patients in effective group and 128 (50.2%) patients in noneffective group. Three (2.3%) of the cases in the noneffective group developed symptomatic intracranial hemorrhage but none in the effective group after IV-rtPA (*P* = .247). One (0.8%) patient in the effective group and 4 (3.1%) patients in the noneffective died within 90 days after IV-rtPA (*P* = .370).

**Table 1:** Baseline characteristics of patients enrolled in the current study and some other important studies.

Factors	Current study	NINDS I	NINDS II	ECASSIII	SITS-MOST
	(n = 255)	(n = 144)	(n = 168)	(n = 418)	(n = 6483)
Male [n (%)]	164 (64.3)	102 (70.8)	107 (63,7)	264 (63)	3902 (60.2)
Age (yr, mean ± SD)	66.89 ± 11.89	67 ± 10	66 ± 11	64.9 ± 12.2	68 (59–75)†
Hypertension history [n (%)]	176 (69.0)	95 (66)	113 (67)	261 (62)	3710 (58.7)
Diabetes history [n (%)]	71 (27.8)	35 (24)	34 (20)	62 (15)	1020 (16)
Current smoking [n (%)]	37 (14.5)	25 (17)	20 (12)	32 (8)	643 (10.1)
CHD history [n (%)]	48 (18.8)	26 (18)	40 (24)	–	–
AF history [n (%)]	55 (21.6)	26 (18)	34 (20)	53 (13)	1507 (23.9)
Liver dysfunction [n (%)]	25 (9.8)	–	–	–	–
Smoking history [n (%)]	106 (41.6)	62 (43)	45 (27)	214 (52)	2643 (43.2)
Drinking history [n (%)]	68 (26.7)	–	–	–	–
Use of antiplatelet drugs history [n (%)]	105 (41.2)	-(41)	-(40)	130 (31.1)	–
Use of antihypertensive drugs history [n (%)]	128 (50.2)	–	–	–	–
Use of hypolipidemic drugs history [n (%)]	96 (37.7)	–	–	–	–
Use of hypoglycemic drugs history [n (%)]	50 (19.6)	–	–	–	–
TOAST					
LAA [n (%)]	73 (28.6)	50 (35)	66 (39)	–	2279 (35.1)
SAO [n (%)]	126 (49.4)	27 (19)	24 (14)	–	535 (8.3)
CE [n (%)]	54 (21.2)	61 (42)	76 (45)	–	2270 (35)
Other [n (%)]	2 (0.8)	4 (3)	3 (2)	–	1171 (18.1)
LDL (mmol/L, mean ± SD)	3.22 ± 0.99	–	–	–	–
FIB (g/L, mean ± SD)	2.88 ± 0.96	3.32 ± 0.94	3.11 ± 1.02	–	–
HCY (μmol/L, mean ± SD)	15.17 ± 8.13	–	–	–	–
Glucose (mmol/L, mean ± SD)	7.84 ± 3.53	8.3 ± 4.2	8.3 ± 3.7	–	6·4 (5·6–7·7)†
SBP (mm Hg, mean ± SD)	152.05 ± 20.08	155 ± 22	153 ± 22	152.6 ± 19.2	150 (137–166)†
DBP (mm Hg, mean ± SD)	84.23 ± 11.57	85 ± 12	85 ± 14	84.4 ± 13.5	81 (74–90)†
OTT (min, mean ± SD)	177.10 ± 74.02	–	–	239 (225–255)†	140 (115–165)†
0–90 min [n (%)]	10 (3.9)	71 (49.0)	86 (51.0)	–	671 (10.6%)
91–180 min [n (%)]	133 (52.2)	73 (51.0)	82 (49.0)	–	4276 (66%)
181–270 min [n (%)]	95 (37.3)	–	–	405 (96.9)	–
>270 min [n (%)]	17 (6.7)	–	–	–	–
NIHSS at baseline (mean ± SD)	9.34 ± 6.92	14 (1–37)*	14 (2–37)*	10.7 ± 5.6	–
Dose (mg/kg, mean ± SD)	0.79 ± 0.13	0.9	0.9	0.9	0.9

The 4 studies, including NINDS I, NINDS II, ECASSIII, and SITS-MOST, were presented for context only.

Data were expressed as mean ± standard deviation (mean ± SD) or n (%).

AF = atrial fibrillation, CE = cardioembolism, CHD = coronary heart disease, DBP = diastolic pressure, ECASSIII = European Cooperative Acute Stroke Study part III, FIB = fibrinogen, HCY = homocysteine, LAA = large artery atherosclerosis, LDL = low-density lipoprotein, NIHSS = National Institute of Health Stroke Scale, NINDS = The National Institute of Neurological Disorders and Stroke, OTT = onset-to-treatment time, SAO = small artery occlusion, SBP = systolic pressure, SITS-MOST = Safe Implementation of Thrombolysis in Stroke-Monitoring Study, TOAST = Trial of Org 10 172 in acute stroke treatment.

* Data were expressed as median (range).

† Data were expressed as median (interquartile range).

### 3.2. Effects of IV-rtPA on OTT and NIHSS

**Figure 1.**
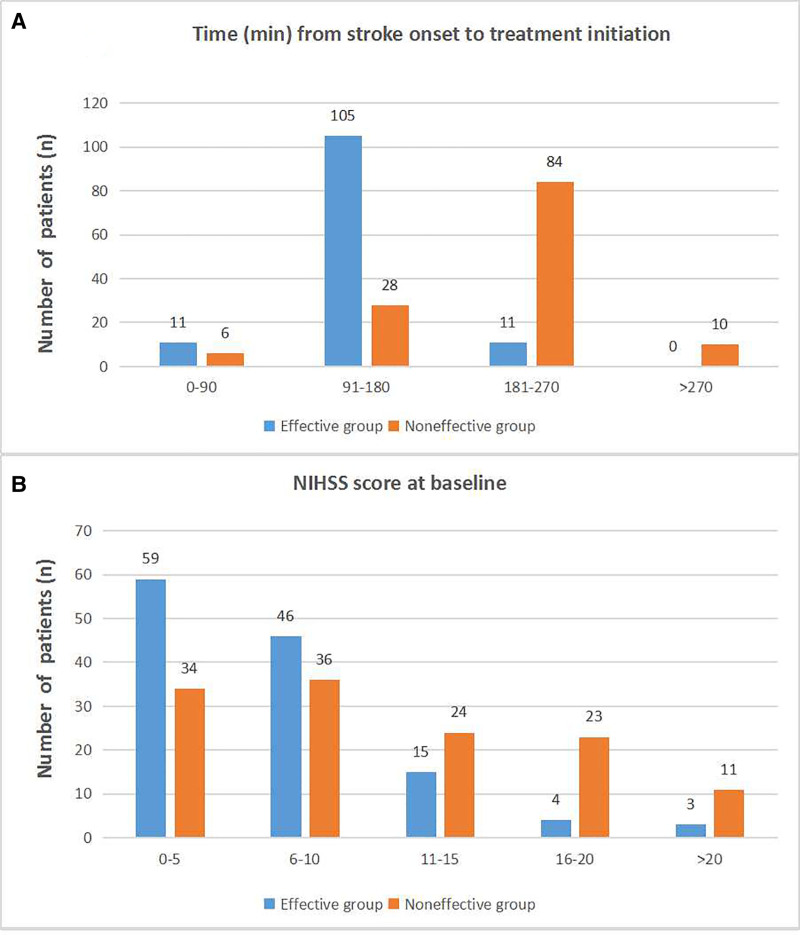
Effect of IV-rtPA on OTT and NIHSS at baseline. (A) Early effect of IV-rtPA on OTT time. (B) Early effect of IV-rtPA on NIHSS. IV-rtPA = recombinant tissue plasminogen activator, NIHSS = National Institute of Health Stroke Scale, OTT = onset-to-treatment time.

Twenty-four hours after thrombolysis with IV-rtPA, 127 of 255 (49.8%) patients reached the early effective standard. Figure 1 showed the early clinical effect of IV-rtPA varied according to OTT time (Fig. 1A) and baseline NIHSS score (Fig. 1B). Further analysis showed that the outcome of IV-rtPA within 3 hours from the onset of AIS was better than that of patients who started IV-rtPA 3 hours after the onset of AIS (*χ*^2^ = 110.434, *P* < .001) (Table 2). Patients with baseline NIHSS score <10 benefited more from IV-rtPA than those with baseline NIHSS score more than 10 (*χ*^2^ = 23.196, *P* < .001) (Table 3).

**Table 2:** Outcomes of the IV-rtPA effect in AIS patients in different time windows [n (%)].

OTT time	Effective rate	Noneffective rate	*χ* ^2^	*P* value
≤3 h (n = 150)	116 (77.3)	34 (22.7)	110.434	<.001
>3 h (n = 105)	11 (10.5)	94 (89.5)		

IV-rtPA = recombinant tissue plasminogen activator, OTT = onset-to treatment.

**Table 3:** Outcomes of the IV-rtPA effect in AIS patients with different baseline NIHSS scores [n (%)].

NIHSS at baseline	Effective rate	Noneffective rate	*χ* ^2^	*P* value
≤10 (n = 175)	105 (60.0)	70 (40.0)	23.196	<.001
>10 (n = 80)	22 (27.5)	58 (72.5)		

IV-rtPA = recombinant tissue plasminogen activator, NIHSS = National Institute of Health Stroke Scale.

### 3.3. Effects of IV-rtPA on mRS scores

**Figure 2.**
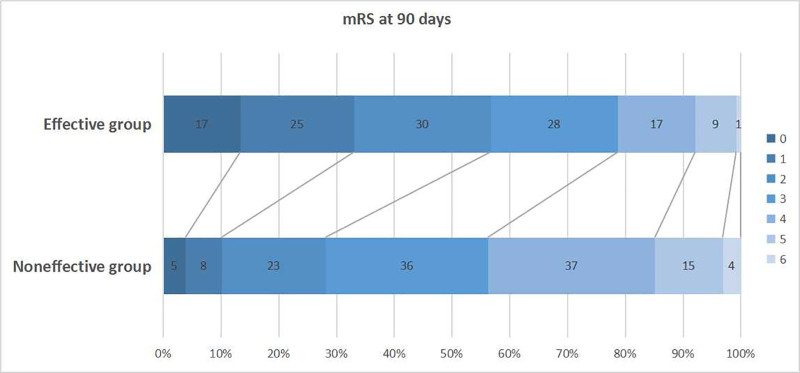
Effect of IV-rtPA on mRS scores at 90 days. IV-rtPA = recombinant tissue plasminogen activator, mRS = modified Rankin Scale.

As shown in Table 4 and Figure 2, the mRS = 0 to 1 (*χ*^2^ = 19.787, *P* < .001) and mRS = 0 to 2 (*χ*^2^ = 11.046, *P* = .001) ratio at 90 days after thrombolysis were significantly higher in effective group than those in noneffective group.

**Table 4:** Outcomes of the mRS at 90 days [n (%)].

Outcomes	Effective group (n = 127)	Noneffective group (n = 128)	*χ* ^2^	*P* value
mRS = 0–1	42 (33.1)	13 (10.2)	19.787	<.001
mRS = 0–2	72 (56.7)	46 (35.9)	11.046	.001

mRS = modified Rankin scale.

### 3.4. Factors influencing the early effect of thrombolysis

Univariate analysis showed there were statistical differences between the 2 groups in gender (*P* = .030), age (*P* < .001), AF history (*P* = .024), drinking history (*P* = .026), FIB level (*P* = .012), SBP level (*P* = .046), OTT time (*P* < .001), baseline NIHSS (*P* < .001), and dose of rtPA (*P* = .012) between the 2 groups (Table 5). Multivariate logistic regression analysis showed the male gender (OR: 3.986, 95% CI: 1.727–9.201, *P* = .001), drinking history (OR: 0.253, 95% CI: 0.124–0.671, *P* = .004), OTT (OR: 0.958, 95% CI: 0.948–0.969, *P* < .001), baseline NIHSS scores (OR: 0.838, 95% CI:0.785–0.895, *P* < .001), and dose of rtPA (OR: 385.527, 95% CI: 16.725–8886.741, *P* < .001) were independently related to the early efficacy, those with male gender, no drinking history, shorter OTT time, lower baseline NIHSS score, and larger dose of rtPA were more likely to exhibit early clinical improvement (Table 6).

**Table 5:** Univariate analysis of factors influencing the early clinical effect of IV-rtPA in the treatment of acute cerebral infarction.

Factors	Groups		*χ*^2^/t	*P* value
	Effective (n = 127)	Noneffective (n = 128)		
Male [n (%)]	90 (70.9)	74 (57.8)	4.733	.030
Age (yr, mean ± SD)	64.17 ± 11.59	69.58 ± 11.61	3.720	<.001
Hypertension history [n (%)]	82 (64.6)	94 (73.4)	2.346	.126
Diabetes history [n (%)]	28 (22.0)	43 (34.0)	0.706	.401
Stroke history [n (%)]	19 (15.0)	18 (14.1)	0.041	.839
CHD history [n (%)]	23 (18.1)	25 (19.5)	0.084	.772
AF history [n (%)]	20 (15.8)	35 (27.3)	5.067	.024
Liver dysfunction [n (%)]	13 (10.2)	14 (10.9)	0.033	.856
Smoking history [n (%)]	55 (43.3)	51 (39.8)	0.033	.575
Drinking history [n (%)]	26 (20.5)	42 (32.8)	4.964	.026
Use of antiplatelet drugs history [n (%)]	50 (39.4)	55 (43.0)	0.341	.559
Use of antihypertensive drugs history [n (%)]	62 (48.8)	66 (51.6)	0.192	.661
Use of hypolipidemic drugs history [n (%)]	46 (36.2)	50 (39.1)	0.219	.640
Use of hypoglycemic drugs history [n (%)]	24 (18.9)	26 (20.3)	0.081	.776
TOAST			8.306	.081
LAA [n (%)]	37 (29.1)	36 (28.1)		
SAO [n (%)]	70 (55.1)	56 (43.8)		
CE [n (%)]	19 (15.0)	35 (27.3)		
Other [n (%)]	1 (0.8)	1 (0.8)		
LDL (mmol/L, mean ± SD)	3.22 ± 1.04	3.21 ± 0.95	-0.100	.92
FIB (g/L, mean ± SD)	2.73 ± 0.88	3.03 ± 1.01	2.518	.012
HCY (μmol/L, mean ± SD)	15.18 ± 7.92	15.15 ± 8.36	-0.031	.975
Glucose (mmol/L, mean ± SD)	7.53 ± 2.94	8.14 ± 4.02	1.379	.169
SBP (mm Hg, mean ± SD)	149.54 ± 17.57	154.55 ± 22.09	2.001	.046
DBP (mm Hg, mean ± SD)	83.91 ± 10.52	84.55 ± 12.55	0.437	.663
OTT (minutes, mean ± SD)	142.22 ± 35.50	211.70 ± 85.34	8.500	<.001
NIHSS at baseline (mean ± SD)	7.45 ± 5.64	11.21 ± 7.56	4.506	<.001
Dose (mg/kg, mean ± SD)	0.82 ± 0.12	0.77 ± 0.14	-2.538	.012

Data were expressed as mean ± standard deviation (mean ± SD) or n (%).

AF = atrial fibrillation, CE = cardioembolism, CHD = coronary heart disease, DBP = diastolic pressure, FIB = fibrinogen, HCY = homocysteine, IV-rtPA = recombinant tissue plasminogen activator, LAA = large artery atherosclerosis, LDL = low-density lipoprotein, NIHSS = National Institute of Health Stroke Scale, OTT = onset-to-treatment, SAO = small artery occlusion, SBP = systolic pressure, TOAST = Trial of Org 10 172 in acute stroke treatment.

**Table 6:** Multivariate logistic regression analysis of factors influencing the early clinical effect of IV-rtPA in the treatment of acute cerebral infarction.

Variables	Odds ratio	95% CI	*P* value
Male	3.986	1.727–9.201	.001
Drinking history	0.253	0.124–0.671	.004
OTT	0.958	0.948–0.969	<.001
NIHSS at baseline	0.838	0.785–0.895	<.001
Dose	385.527	16.725–8886.741	<.001
Constant	40.706		.009

IV-rtPA = recombinant tissue plasminogen activator, NIHSS = National Institute of Health Stroke Scale, OTT = onset-to-treatment.

## 4. Discussion

In the current study focusing on the cohort of 255 AIS patients treated with IV-rtPA, it is demonstrated that approximately half of AIS patients treated with IV-rtPA achieved early neurological improvement within 24 hours, and nearly half attained functional independence by 90 days. Importantly, we identified 5 independent factors that significantly influenced the early efficacy of thrombolysis: male gender, absence of drinking history, shorter OTT, lower baseline NIHSS score, and higher rtPA dose. Beyond describing these clinical outcomes, our primary objective was to identify the determinants of these outcomes. Multivariate logistic regression analysis demonstrated that male gender, absence of drinking history, shorter OTT, lower baseline NIHSS score, and higher rtPA dose were independently associated with a greater likelihood of early neurological improvement. These findings demonstrate that early neurological improvement at 24 hours predicts favorable long-term functional recovery and that the safety profile of alteplase in our real-world cohort is consistent with established clinical benchmarks. Compared with landmark randomized trials establishing benchmarks for both efficacy and safety of IV alteplase in AIS, including NINDS I, NINDS II, ECASSIII, and SITS-MOST,^[15–18]^ our achieved comparable rates of neurological recovery, functional independence, and low sICH incidence. However, our analysis extends these findings by linking them to concrete predictors of efficacy: for example, patients with lower baseline NIHSS scores and shorter OTT times not only had higher rates of early neurological recovery but also better long-term functional outcomes. Conversely, patients with a history of alcohol consumption or higher baseline stroke severity demonstrated poorer responses, underscoring the importance of individualized risk stratification when considering thrombolytic therapy. These findings underscore that the clinical benefits and risks of IV-rtPA are not solely determined by the therapy itself but are strongly modulated by patient characteristics and treatment-related variables, highlighting the importance of individualized treatment strategies in AIS management.

The OTT is undeniably one of the most crucial factors influencing the effectiveness of thrombolytic therapy. The brain is an organ with high oxygen consumption and minimal oxygen reserves. Even brief ischemia can lead to irreversible brain damage. Therefore, promptly reopening any occlusion and restoring blood supply to the ischemic area is critically important. Studies have demonstrated that IV-rtPA is the most effective method for achieving this, with the benefits being greater the earlier it is administered.^[21]^ Research by Saver et al found that shorter OTT times were associated with lower in-hospital mortality and symptomatic intracranial hemorrhage, as well as higher rates of discharge to home and independent ambulation at discharge.^[22]^ While some studies have shown that IV-rtPA can be effective up to 6 hours after the onset of AIS,^[23]^ most research indicates that the optimal window for administration is within 4.5 hours, with the most significant effects observed when given within 3 hours.^[24,25]^ Recent studies, such as the WAKE UP^[26]^ and EXTEND^[27]^ trials, have explored the use of tissue window rather than time window, including patients with unclear onset times and those beyond the traditional thrombolysis window but with significant ischemic penumbra. These studies achieved positive outcomes, suggesting that while the time window remains critical, expanding it using tissue criteria can also be effective.

In our current study, we observed that the OTT time in the effective group was significantly shorter than in the noneffective group. Further analysis revealed that IV-rtPA thrombolysis within 3 hours of AIS onset provided more benefits than beyond 3 hours. Multivariate logistic regression analysis indicated that OTT time was independently related to the early clinical effect of IV-rtPA. Prior research^[28]^ has identified various factors influencing OTT time, including out-of-hospital factors like patient and family awareness, mode of hospital arrival, and traffic conditions, as well as in-hospital factors such as physician attention to OTT time, consent process duration, necessary examinations, and thrombolysis execution speed. Thus, enhancing public awareness of AIS thrombolysis and streamlining thrombolysis procedures to create a “green channel” for rapid treatment is essential to improve outcomes.

Baseline NIHSS scores of patients also significantly impact the clinical effectiveness of thrombolysis. Studies have shown that a high baseline NIHSS score is an independent risk factor for poorer thrombolytic outcomes and symptomatic intracranial hemorrhage.^[9,29]^ In our study, the effective group had lower baseline NIHSS scores compared to the noneffective group. Patients with baseline NIHSS scores <10 benefited more from IV-rtPA, and the ratio of mRS scores of 0 to 1 and 0 to 2 at 90 days post-thrombolysis was significantly higher in the effective group. Multivariate logistic regression analysis confirmed that a lower baseline NIHSS score was independently related to early clinical improvement. Therefore, for AIS patients with high NIHSS scores, it is crucial to promptly evaluate the suitability for intravenous thrombolysis and consider alternative interventions like interventional thrombectomy or intravascular thrombolysis if indicated.

The dose of thrombolytic drugs is another vital factor affecting thrombolysis outcomes. The optimal dose for treating AIS remains controversial. Based on several large international studies, current guidelines recommend a standard rtPA dose of 0.9 mg/kg (maximum 90 mg).^[25]^ However, Japanese studies have recommended a lower dose of 0.6 mg/kg.^[30]^ A 2016 international multi-center clinical study showed that the 0.6 mg/kg dose did not achieve the same clinical efficacy as 0.9 mg/kg but did reduce the incidence of symptomatic intracranial hemorrhage.^[31]^ Our study found that the effective group received a higher dose of rtPA, and multivariate logistic regression analysis indicated that the dose was an independent factor affecting thrombolytic efficacy. Further research is needed to determine the optimal dose in conjunction with the overall patient condition.

Alcohol consumption is closely linked to stroke risk. Studies have shown that mild to moderate alcohol consumption increases the relative risk of stroke and ischemic stroke.^[31]^ Another survey found that even low-dose alcohol consumption significantly increases ischemic stroke risk.^[32]^ Long-term follow-up studies have shown that chronic alcohol consumption leads to higher stroke-related mortality.^[33]^ In our study, a higher proportion of patients in the noneffective group had a history of alcohol consumption. Multivariate logistic regression analysis confirmed that drinking history was an independent factor affecting thrombolytic efficacy. This may be due to alcohol’s effects on nerve cell necrosis, nutrient absorption impairment, vascular endothelial damage, blood pressure increase, and accelerated atherosclerosis.^[34]^ Gender also influences thrombolytic therapy efficacy, with female patients generally benefiting less. This may be due to higher rates of metabolic syndrome and thrombolytic drug resistance in females. Studies have found higher levels of plasminogen activator inhibitor-1 in female AIS patients, which is an independent predictor of thrombolysis resistance.^[35]^ In addition to the aforementioned factors, our univariate analysis found significant differences in age, atrial fibrillation history, fibrinogen level, and systolic blood pressure between the effective and noneffective groups. However, these factors did not show statistical significance in multivariate logistic analysis, possibly due to the study small sample size and retrospective nature.

## 5. Conclusions

In summary, this retrospective analysis of 255 patients with AIS demonstrated that the early efficacy of intravenous thrombolysis with alteplase is significantly influenced by both patient- and treatment-related factors. Male gender, absence of drinking history, shorter onset-to-treatment time, lower baseline NIHSS score, and higher rtPA dose were identified as independent predictors of favorable early neurological improvement, while age, atrial fibrillation, elevated fibrinogen levels, and higher systolic blood pressure may also contribute as potential risk factors. These findings not only reaffirm the importance of timely and appropriately dosed IV-rtPA but also highlight the need for individualized patient assessment to optimize therapeutic benefit and minimize risks. Future large-scale prospective studies are warranted to validate these associations and to refine clinical decision-making algorithms for stroke thrombolysis.

## Author contributions

**Conceptualization:** Yunyun Zhang.

**Data curation:** Jiwei Cheng, Zhen Yuan, Yunqing Zeng, Yangbo Hou, Yunyun Zhang.

**Formal analysis:** Jiwei Cheng, Zhen Yuan, Yunqing Zeng, Yangbo Hou, Yunyun Zhang.

**Writing – original draft:** Jiwei Cheng.

**Writing – review & editing:** Yangbo Hou, Yunyun Zhang.
